# Histamine Modulation of the Basal Ganglia Circuitry in the Motor Symptoms of Parkinson's Disease

**DOI:** 10.1111/cns.70308

**Published:** 2025-02-27

**Authors:** Hui‐Xian Zhu, Wei‐Wei Lou, Yi‐Miao Jiang, Alina Ciobanu, Chen‐Xin Fang, Cheng‐Ye Liu, Yan‐Li Yang, Jing‐Yang Cao, Ling Shan, Qian‐Xing Zhuang

**Affiliations:** ^1^ Department of Physiology, School of Medicine Nantong University Nantong Jiangsu China; ^2^ Department of Neuropsychiatric Disorders Netherlands Institute for Neuroscience, an Institute of the Royal Netherlands Academy of Arts and Sciences Amsterdam the Netherlands

**Keywords:** basal ganglia, histamine, histamine receptor, motor symptoms, Parkinson's disease

## Abstract

**Purpose of Review:**

Parkinson's disease (PD) is characterized by dopaminergic system dysfunction that results from the degeneration of neurons in the substantia nigra. However, studies suggest that other neurotransmitters, especially histamine, may also play a role in the development of PD.

**Recent Findings:**

Numerous studies show that histamine levels in the basal ganglia significantly change in PD pathology, correlating with motor symptoms observed in animal models of PD. Histamine activates H1R or H4R on microglia in the substantia nigra, triggering an inflammatory response and promoting dopaminergic neuron degeneration. Additionally, histamine modulates neuronal excitability and firing activity (firing rate and pattern) by activating H1R, H2R, or H3R on neurons in the basal ganglia nucleus, ultimately impacting normal motor behavior as well as motor symptoms in models of PD.

**Summary:**

This review presents the role of histamine and its receptor ligands in the basal ganglia nuclei, along with downstream ion channels linked to histamine receptors that influence immune response, neuronal excitability, and firing activity in PD. It highlights their effects on neuronal firing and their connection to PD motor symptoms. Investigating new ligands targeting basal ganglia histamine receptors and associated ion channels may facilitate the development of novel treatments for PD.

## Graphical abstract

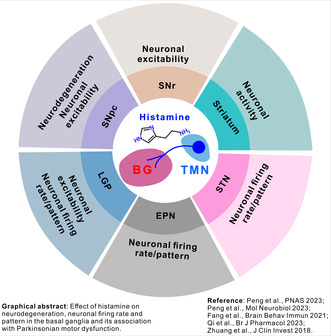

**Abbreviations**
6‐OHDA: 6‐hydroxydopamine
BBB: Blood‐brain barrier
cAMP: cyclic adenosine monophosphate
DBS: Deep brain stimulation
d‐SPNs: Direct pathway‐striatal projection neurons
EEG: Electroencephalogram
EPN: Entopeduncular nucleus
GANA: Gamma‐aminobutyric acid
GPCR: G protein‐coupled receptor
GPe: Globus pallidus externa
GPi: Globus pallidus interna
GRK3: G‐protein‐coupled receptor kinases 3
H1R: Histamine H1 receptor
H2R: Histamine H2 receptor
H3R: Histamine H3 receptor
H4R: Histamine H4 receptor
HCN: Hyperpolarization‐activated cyclic nucleotide‐gated
IL‐1β: Interleukin‐1β
IL‐6: Interleukin‐6
i‐SPNs: Indirect pathway‐striatal projection neurons
LGP: Lateral globus pallidus
LID: L‐dopamine‐induced dyskinesia
NO: Nitric oxide
NOX1: NADPH oxidase 1
PD: Parkinson's disease
PS: Phosphatidylserine
PV: Parvalbumin
ROS: Reactive oxygen species
SK3: Small‐conductance calcium‐activated potassium channel 3
SN: Substantia nigra
SNpc: Substantia nigra pars compacta
SNr: Substantia nigra pars reticulata
Sst: Somatostatin
STN: Subthalamic nucleus
TMN: Tuberomammillary nucleus
TNF‐α: Tumor necrosis factor‐α
TREK‐1: TWIK‐related potassium channel‐1
α‐FMH: Alpha‐fluoromethylhistidine

## 1 Introduction

The basal ganglia is a vital motor regulatory structure positioned beneath the cortex that connects the cerebral cortex, thalamus, cerebellum, hippocampus, amygdala, and motor nuclei of the brainstem [1]. The aforementioned structure is responsible for the regulation of voluntary physical movements and muscular tension. Additionally, it is involved in the processing of proprioceptive afferent impulses, achieved by linkages between its afferent and efferent nuclei [2, 3]. It is widely accepted that motor function regulation in the basal ganglia is accomplished through three primary pathways: the direct, indirect, and hyper‐direct pathways. Normally, the direct and indirect pathways within the basal ganglia circuitry maintain a complex equilibrium, regulated by the hyper‐direct pathway originating from the cerebral cortex. This intricate balance is essential for ensuring proper and random body movement [4, 5, 6, 7]. However, when this balance is disrupted, the body suffers from various motor disorders, including Parkinson's disease (PD) and Huntington's disease [8, 9, 10].

Although the central neuronal histaminergic system originates in the tuberomammillary nucleus (TMN) of the hypothalamus, its fibers project extensively to various regions of the brain, including the basal ganglia [11, 12, 13]. Patients with PD exhibit specific functional changes in the histaminergic system, as confirmed by a significant increase in histamine levels across all basal ganglia nuclei of postmortem human PD brains. Among them, histamine levels in the putamen increased to 159%, the substantia nigra pars compacta (SNpc) increased to 201%, the globus pallidus interna (GPi) increased to 234%, and the globus pallidus externa (GPe) increased to 200% [14]. Consistent with this, histamine fibers were significantly increased in the SNpc of postmortem PD brains [13]. Similarly, studies in rats have shown that elevated histamine levels in the SN may lead to degeneration of dopaminergic neurons [15, 16]. Thus, it is plausible that disruptions to neurotransmitters of the central histaminergic system and their functions are intricately linked to the manifestation of motor symptoms in PD.

PD is the second most prevalent neurodegenerative disorder globally, severely impacting patients' quality of life and exerting significant mental and economic strain on their families [17]. Patients diagnosed with PD commonly present with motor manifestations such as resting tremors, rigidity, and bradykinesia. Additionally, they may experience non‐motor symptoms, including sleep disturbances, impairments in learning and memory, cognitive decline, and depressive symptoms [18]. Despite the fact that the mechanisms underlying the progression of PD pathology remain incompletely understood, the degeneration and programmed cell death of substantia nigra (SN) dopaminergic neurons, which play a crucial role in regulating the striatum and other basal ganglia nuclei, have been identified as key pathological mechanisms underlying the symptoms of PD [19, 20]. Correspondingly, the primary pharmacological approach for managing PD involves the oral administration of levodopa to replenish striatal dopamine levels [21]. However, existing evidence suggests that the extended administration of oral levodopa to individuals with PD may lead to the occurrence of “on–off” phenomena and movement problems, such as levodopa‐induced dyskinesia (LID) [22, 23, 24]. Moreover, the likelihood of experiencing adverse effects is heightened in this context [25]. Finding a safer and more effective treatments for PD patients is therefore crucial, and developing new drugs without side effects has become a global challenge. The glutamatergic, GABAergic, cholinergic, noradrenergic, and histaminergic neurotransmitter systems that innervate the basal ganglia circuits all have significant involvement in the pathogenesis of PD [26, 27, 28, 29]. In recent years, there has been a growing body of research focused on investigating the impact of histamine on PD and other neurological systems [20, 30, 31]. In this review, the aim was to summarize how histamine interacts with important nuclei in the basal ganglia and its effects on motor symptoms in individuals with PD.

## 2 Basal Ganglia Function

### 2.1 Basal Ganglia and Its Components

The basal ganglia consist mainly of the striatum, globus pallidus (GP), subthalamic nucleus (STN) and SN. Additionally, SN can be further divided into SNpc and substantia nigra pars reticular (SNr). In primates, the striatum can be further divided into caudate nucleus and putamen, and GP is divided into GPe and GPi, while in rodents, GPe and GPi are usually referred to as the lateral globus pallidus (LGP) and entopeduncular nucleus (EPN) [32]. Anatomically, the basal ganglia are situated between the telencephalon and the midbrain, with the main structures, such as the GP and striatum, located in the telencephalon [33]. The basal ganglia structure consists of three parts: input core, output core, and intermediate core. The input nuclei comprise the striatum and STN, which receive inputs from the cerebral cortex, thalamus, and SNpc. The intermediate nucleus serves as a relay nucleus between the input and output nuclei, predominantly comprising the LGP. The efferent nucleus comprises the EPN and the SNr. The output nuclei exert regulatory influence on the thalamus, which in turn transmits signals to the cortex [34]. The aforementioned neural circuitry creates a closed loop consisting of the cortex, basal ganglia, thalamus, and cortex. This loop serves to facilitate the control of motor information transmission [35].

### 2.2 Role of the Basal Ganglia in Motor Control

The basal ganglia modulate motor information from the cortex via its two internal pathways, the direct and indirect pathways [36]. Histological evidence shows that the striatum region, an essential nucleus of the basal ganglia, has functional zoning and loop characteristics that enable it to integrate with the cerebral cortex [37]. Normal motor ability relies on the stability of the SN‐striatum pathway in the basal ganglia [38]. Under physiological conditions, the concentration of dopamine in the striatum remains stable, and the dopamine receptor is continuously active [39]. Furthermore, the sequential modulation of dopamine release in the SN and the heightened stimulation of dopamine receptors in the striatum contribute to the reward system underlying motor behavior [40]. In pathological conditions, particularly in the pathogenesis of PD, there is a notable increase in the extensive degradation of dopamine fibers [20]. This results in heightened stereotyped behavior and diminished spontaneous movements [41].

Basal ganglia and cerebellum have always been classified as areas solely related to motor regulation [2]. However, with the progress of many advanced technologies such as neuroimaging technology and virus tracing programs, a large number of studies have found that basal ganglia and cerebellum are directly related to the neocortex and the limbic network. These structures actively contribute to the modulation and processing of motor and emotional functions [42, 43]. However, it is important to do a comprehensive investigation of the particular regulatory loop [44]. Moreover, an electroencephalogram (EEG) study has provided evidence supporting the involvement of the striatum in the cognitive regulation process. Specifically, the striatum is shown to indirectly contribute to the management of conflicts arising from the interplay between conscious and subconscious mental processes [45]. In the PD mouse model, functional connectivity between the cerebellar subregion and the basal ganglia is decreased. The decreased connectivity of the STN was associated with motor dysfunction, while the decreased connectivity of the caudate nucleus had a direct impact on cognitive impairment [46].

### 2.3 Intricate Circuitry Within the Basal Ganglia

**FIGURE 1:**
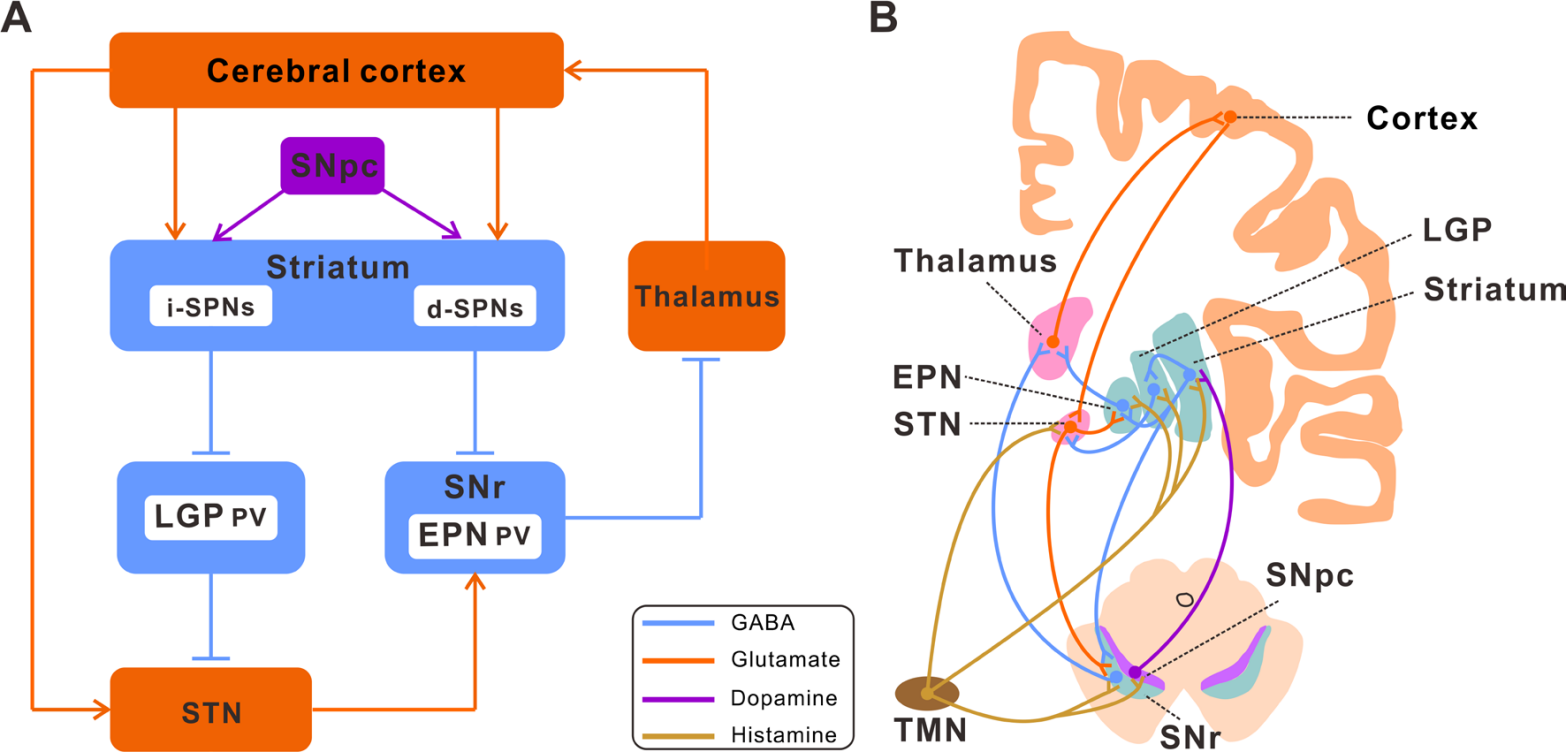
Basal ganglia circuit and histaminergic fibers innervation in the basal ganglia. (A) The direct, indirect and hyper‐direct pathways of the basal ganglia, and the heterogeneity of neurons within the nuclei that compose the basal ganglia, as well as the intricate circuitry connecting them. (B) Histaminergic fibers originating from the neurons of the TMN directly innervate the SNpc, striatum, LGP, EPN, SNr, and STN of the basal ganglia. d‐SPNs, Direct pathway‐striatal projection neurons; EPN, Entopeduncular nucleus; i‐SPNs, Indirect pathway‐striatal projection neurons; LGP, Lateral globus pallidus; PV, Parvalbumin; SNpc, Substantia nigra pars compacta; SNr, Substantia nigra pars reticulata; STN, Subthalamic nucleus; TMN, Tuberomammillary nucleus.

The basal ganglia motion control loop is composed of three main pathways: the direct pathway, the indirect pathway, and the hyper‐direct pathway (Figure 1A). Under normal circumstances, dopaminergic neurons of the SN play significant roles in both positively and negatively regulating striatal projection neurons. These projection neurons serve as the origin for the direct and indirect pathways, respectively [6]. Through these two pathways, the basal ganglia control the excitatory output of the thalamus to the premotor cortex and facilitate the timely release of motor programs [36]. In the pathological process of PD, the degeneration of dopaminergic neurons in the SN leads to an imbalance in these pathways [47]. This imbalance prevents the normal release of motor programs, causing PD dyskinesia. Interestingly, researchers have offered two models—the antagonistic model and the synergistic model—to explain how these two pathways operate in normal basal ganglia functioning. According to the traditional antagonistic model, the direct pathway facilitates the generation of planned movements, whereas the indirect pathway reduces the generation of planned movements [48, 49]. The synergistic model posits that the direct pathway facilitates the production of anticipated movement, whereas the indirect pathway restrains competitive movements that are irrelevant to the intended goal [50, 51].

However, the classical model encounters some paradoxical challenges. For example, research investigations have revealed that the activation of D1 and D2 receptors (D1R and D2R) inside the striatum is not exclusively associated with excitation and inhibition, nor does it exhibit any discernible connection to the direct and indirect pathways [50, 52]. Furthermore, via continued investigation of the basal ganglia loop, an increasing number of remarkable regulatory loops have been identified. The neurons in the motor and non‐motor domains of the cerebellar dentate nucleus connect to the sensorimotor and associative regions in the striatum through the thalamic nucleus [53]. The impairment of motor learning, which is facilitated by the cortical striatal loop, is influenced by cerebellar damage. Additionally, the presence of tremors in the PD mouse model is also associated with this loop [54]. The striatum, serving as the principal core of the basal ganglia, operates via three primary circuits. Firstly, the putamen sensorimotor circuit transmits its output to the primary motor cortex, auxiliary motor area, and premotor cortex. Secondly, the caudate nucleus combined circuit projects its output to the prefrontal cortex. Lastly, the ventral striatum marginal circuit directs its output to the anterior cingulate cortex and medial prefrontal cortex [41]. Therefore, one should not simply regard the cortical basal ganglia thalamic cortical loop as an independent structure involved in motor regulation but instead consider the interactions between other nuclei involved in motor control associated with the basal ganglia (such as the cerebellum) as well. Hence, there is a need for further evidence in order to enhance the understanding of the basal ganglia loop.

## 3 Histaminergic Inputs in the Basal Ganglia

### 3.1 Histaminergic System in the Brain

**FIGURE 2:**
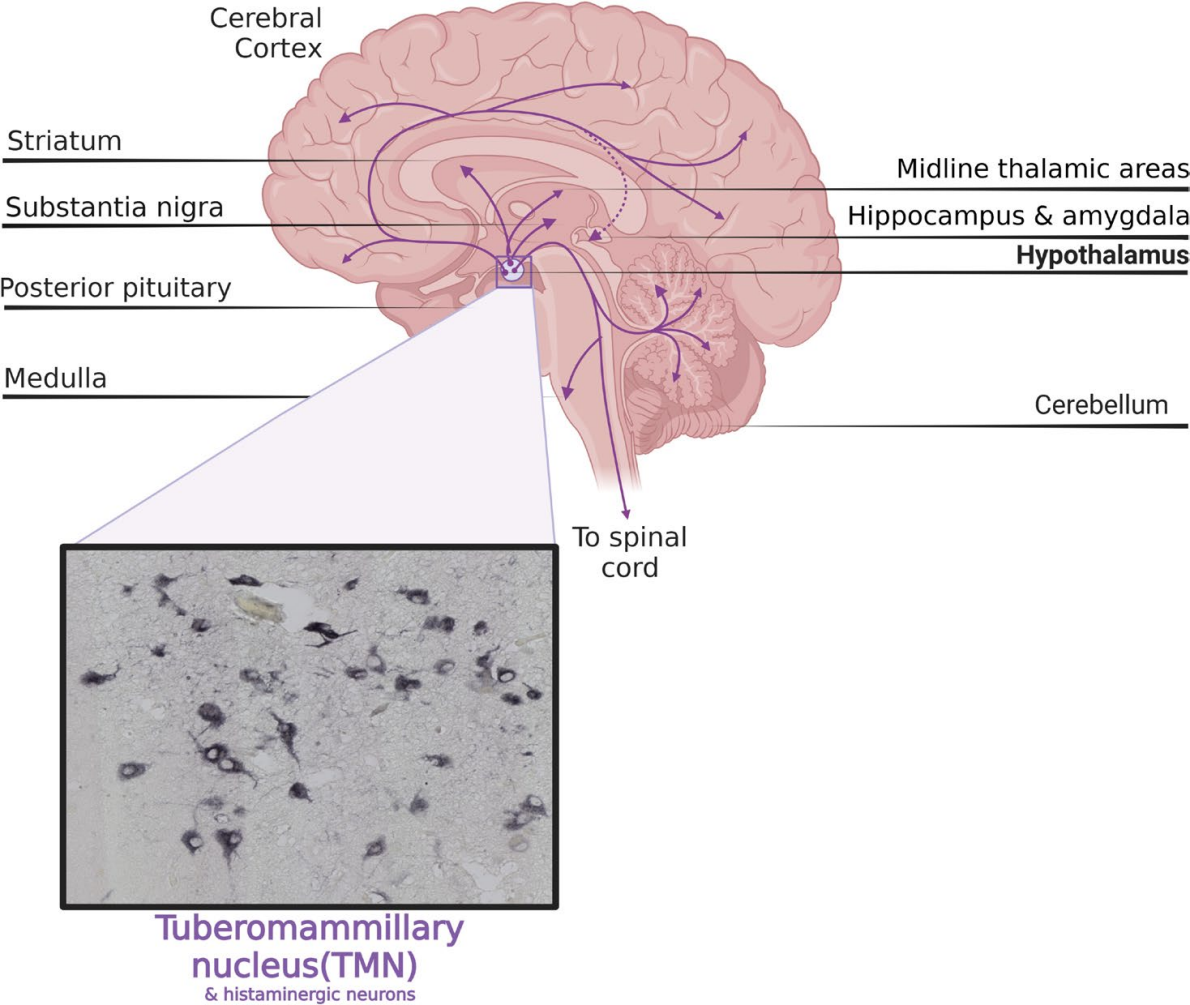
Schematic illustration of the tuberomammillary nucleus (TMN) in the posterior hypothalamus, which sends axonal projections to all areas of the brain including the basal ganglia, where it modulates functions related to motor control. The image shows Histidine decarboxylase (HDC)‐immunoreactive neurons in the TMN with typical TMN cell morphology and long, HDC‐immunoreactive axons (Ms. Alina Ciobanu unpublished observation). [*Created with Biorender.com*].

The primary source of neuronal histamine in the brain is mostly derived from the TMN located in the hypothalamus. Axons originating from the TMN extend widely to other parts of the brain, including the spinal cord [55, 56, 57, 58, 59] (Figure 2). The TMN also receives extensive GABAergic input from brain regions such as the marginal subdermal layer, lateral septum, and preoptic nucleus [60]. In addition, histamine in the brain can also originate from local mast cell secretion in the pia mater, thalamus, and hypothalamus, but its metabolic rate is much slower than that of histaminergic fibers [61]. Previous studies have implicated histamine in the regulation of non‐somatic functions, such as sleep–wake cycles, learning and memory, feeding, energy balance, and temperature regulation [62, 63]. Nevertheless, emerging data indicate that the histamine/central histaminergic nervous system plays a role not only in the control of non‐somatic functions but also in the direct regulation of somatic motor functions via subcortical motor regulatory structures like the cerebellum and basal ganglia [64, 65, 66].

Four types of histamine receptors (H1‐H4R) are present in the vertebrate brain and expressed in specific regions. Histamine receptors H1, H2, and H4R exert their effects on the post‐synaptic cells, while H3R functions as both a pre‐ and post‐synaptic receptor that modulates other neurotransmitter release [67, 68, 69]. All four types of histamine receptors have been identified and isolated, and their gene sequences have been fully elucidated. They belong to the G protein‐coupled receptor (GPCR) family with seven transmembrane regions [55, 56, 70]. Functionally, the activation of H1R and H2R primarily mediates the excitation of neurons despite their opposing effects on calcium ion release [56, 71]. Specifically, H1R promotes the release of arachidonic acid and other substances, whereas H2R inhibits such releases. Consequently, the contrasting effects of H1R and H2R in many tissues can be attributed to this mechanism [60]. The H3R, however, functions pre‐ and post‐synaptically to inhibit the synthesis and release of histamine through feedback. At the same time, it also regulates the release of other neurotransmitters, such as dopamine, glutamate, gamma‐aminobutyric acid (GABA), and acetylcholine, on non‐histaminergic fibers [70]. H3R is expressed mainly in the brain, especially in the cortex, hippocampus, and caudate nucleus, and to a lesser extent in the peripheral areas, followed by the anterior olfactory nucleus, amygdala, striate terminalis, cerebellum, and thalamus [60, 68]. H4R expression is predominantly found in hematopoietic cells [72]. However, recent studies have presumed that it is also functionally expressed in the glial compartment of the brain [30, 31].

### 3.2 Histamine Modulates Basal Ganglia Function

Each basal ganglia constituent nucleus receives input from a variety of histaminergic fibers originating from the TMN (Figure 1B). The striatum, which is the primary input nucleus of the basal ganglia, has a significant number of histamine receptors and also receives projections from histaminergic fibers. The striatum expresses H1R, H2R, and particularly H3R [56]. Numerous studies have provided evidence suggesting that histamine is involved in the regulation of striatal output [73, 74, 75, 76]. Histamine modulates the activity of striatal projection neurons (SPNs) in the striatum by two mechanisms: by activating cholinergic interneurons and by controlling the release of GABA and glutamate, thereby adjusting the output intensity of the striatal circuit. The activation of H3R in the striatum has been seen to result in a significant reduction in GABAergic synaptic transmission by about 50% [77]. This reduction encompasses synaptic transmission originating from other SPNs and inhibitory interneurons within the striatum, as well as synaptic transmission originating from LGP neurons located outside the striatum [78]. Additionally, the activation of H3R can effectively hinder the release of glutamatergic transmitters from the thalamus in the striatum, consequently regulating the excitatory input from the thalamus to the striatum [11, 68]. This suggests that histamine produces an inhibitory impact on the thalamus –striatum input when wakeful or focused. Histamine can also inhibit the lateral feedback inhibition between SPNs without affecting the feedforward inhibition of SPNs mediated by parvalbumin (PV)‐positive fast‐spiking interneurons [79]. The synaptic connection between SPNs plays a crucial role in the plasticity of striatal function and is also an important factor in the regulation of striatal function by histamine [61].

Additional research suggests that the coexistence of H1R and H2R with D1 and D2 receptors in SPNs allows for the direct modulation of SPNs output by the activation of respective post‐synaptic receptors [11, 76]. H1R and H2R are expressed in GABAergic LGP PV neurons. Histamine can directly activate these two post‐synaptic receptors to stimulate PV neurons and impact animal motor behavior by regulating their excitability [12]. Moreover, H2R is also distributed in STN neurons. Histamine facilitates locomotor function in animals through the activation of the hyperpolarization‐activated cyclic nucleotide‐gated (HCN) channel, which is downstream of the H2R receptor [80]. This activation is evidenced by the increased duration of rats on the accelerating rota‐rod and successful traversal of the balancing beam [66]. Furthermore, H2R and H3R are also expressed in EPN, the main output nucleus of the basal ganglia. The motor capacity of mice can be enhanced by pharmacological activation of both these receptors. However, the promotion of this effect is hindered when there is a specific knockout of H2R on EPN PV neurons or pre‐synaptic H3R in STN neurons in its upstream nucleus [20].

## 4 Histaminergic Afferents in Basal Ganglia and PD Motor Symptoms

Histaminergic afferents are known to exert a modulatory influence on the circuitry of the basal ganglia, and their potential involvement in the pathogenesis of PD has been suggested. Previous studies have demonstrated that the basal ganglia receive abundant histaminergic inputs from the hypothalamic TMN and that the density of histaminergic fibers, as well as H3R binding, is significantly greater in the SN of individuals with PD [12, 70, 76]. Despite the current lack of understanding regarding the exact mechanism, it is plausible that histaminergic regulation may exert an influence on the degeneration of dopaminergic cells in the basal ganglia. Moreover, research has found that histaminergic receptor agonists can significantly alleviate motor symptoms in rodent models of PD [20, 66]. These promising findings could potentially have implications for clinical treatment, although further research is needed to fully understand the role of histaminergic afferents in PD pathology (Table 1). Since basal ganglia circuit activity is strongly related to motor dysfunction in PD, the current understanding of basal ganglia histaminergic modulation and its possible relevance to the motor symptoms of PD will be reviewed.

**TABLE 1:** Histaminergic innervation in the basal ganglia nuclei and PD pathology.

Nuclei	HR activation	Effects
SNpc	H1R	Dopaminergic neuronal degeneration [81]
	H1R	Potentiating motor impairment in PD [82]
	H3R	Decreasing the excitability of dopaminergic neurons [83]
	H4R	Dopaminergic neuronal degeneration [31]
SNr	H3R	Enhancing the excitability of dopaminergic neurons [84]
Striatum	H2R	Potentiating the symptoms of LID [85, 86, 87]
	H3R	Reduce involuntary movements [88, 89]
STN	H2R	Regularizing neuronal firing pattern and alleviating motor impairment [66]
	H3R	Decreasing firing rate of EPN PV neurons (receiving STN projections) and alleviating motor impairment [20]
LGP	H1R	Promoting the locomotor augmentation [90]
	H1R	Increasing the excitability of PV neurons [12]
	H1R	Strengthening the induction and expression of exercise sensitization [91]
	H2R	Increasing the excitability of PV neurons [12]
	H3R	Decreasing the excitability of LGP PV neurons [12]
	H3R	Promoting motor impairment [92]
EPN	H2R	Regularizing neuronal firing pattern and alleviating motor impairment [20]

Abbreviations: EPN, entopeduncular nucleus; LGP, lateral globus pallidus; LID, L‐dopamine‐induced dyskinesia; PD, Parkinson's disease; PV, parvalbumin; SNpc, substantia nigra pars compacta; SNr, substantia nigra pars reticulata; STN, subthalamic nucleus.

### 4.1 Histamine in the Substantia Nigra

**FIGURE 3:**
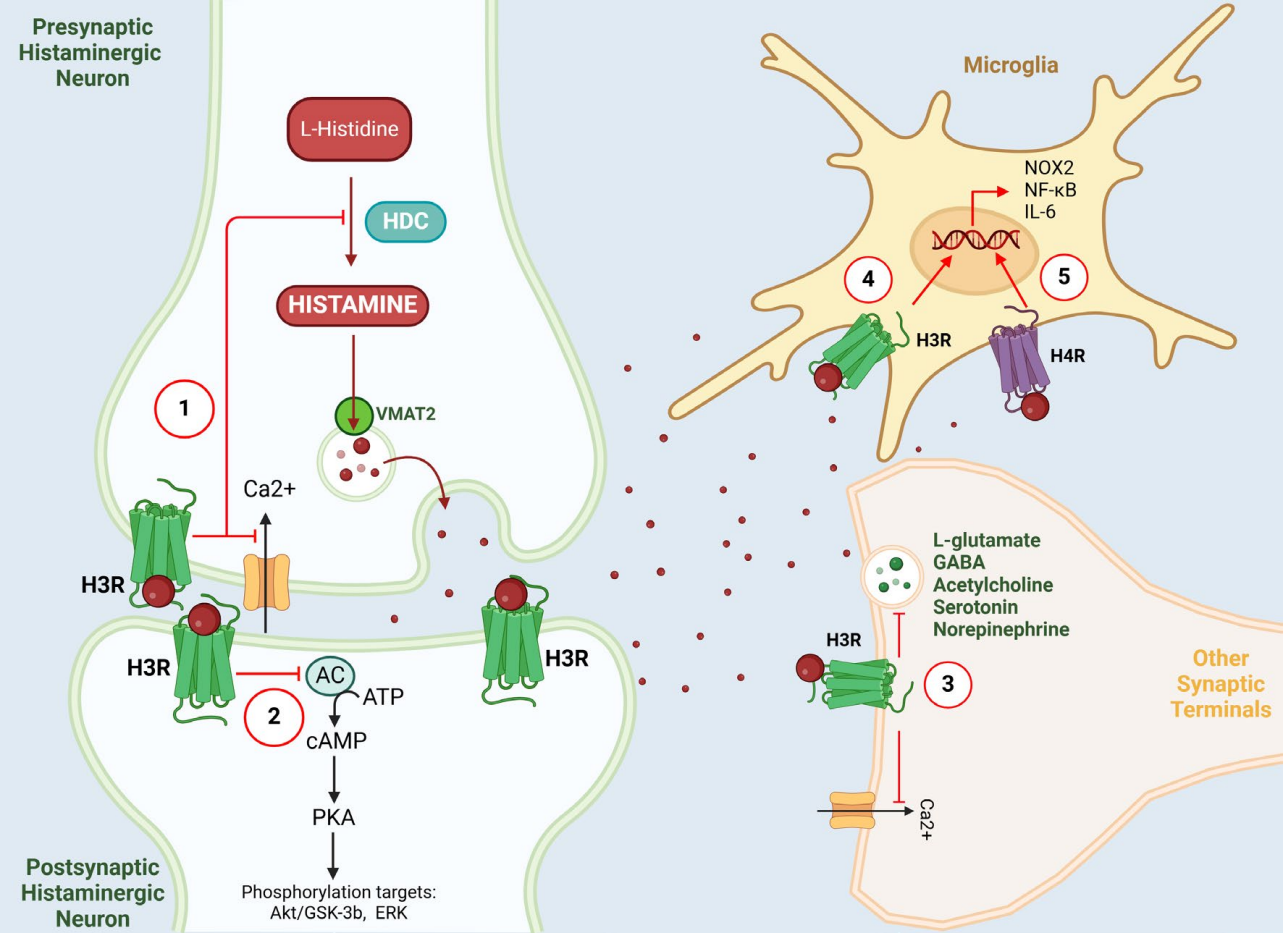
H3R expression in the brain and intracellular pathways regulated by histamine receptors. (1) High‐affinity H3R auto‐receptor inhibits histamine synthesis and release, by inhibiting calcium influx at the pre‐synaptic terminal. HDC, histidine decarboxylase; VMAT, vesicular mono‐amino transporter. (2) H3R auto‐receptor mechanism also inhibits the activity of *adenyl cyclase (AC)*, which in turn activates *phospho‐kinase A (PKA)* and its phosphorylation targets such as *Akt/GSK‐3b* and *ERK* which play important roles in plasticity and various CNS disorders. (3) H3R heteroreceptors on other synaptic terminals, modulate the release of other neurotransmitters such as L‐glutamate and serotonin by inhibiting calcium influx. (4, 5) H3R heteroreceptors and H4R receptors on microglia modulate chemotaxis, phagocytosis and cytokine release functions. [*Created with Biorender.com*].

The SNpc inside the basal ganglia nuclei is recognized as the primary site of pathology in individuals diagnosed with PD. In the SNpc of PD patients, there is an increase in both the projection of histaminergic fibers and the concentration of histamine [14]. The alterations occur in the projection terminals of histaminergic fibers, characterized by a reduction in size and an increase in the size of varicose bodies [13]. A postmortem study observed that in the same brain areas of PD patients, there was an up‐regulation of HMT‐mRNA [83], which may act as a protective mechanism by metabolizing enhanced histamine levels in these areas. Such a protective effect might be of importance since animal experiments have shown that increased histamine levels in the SN may cause microglia activation and dopaminergic neuronal toxicities through H1R rather than H2R [16, 81]. In addition, increased numbers of studies indicate that histamine, acting through its receptors, plays a role in the inflammatory response of PD and the degeneration of dopaminergic neurons [30, 31, 81, 82] (Figure 3). Histamine regulates the migration of N9 microglia and the release of interleukin‐1β (IL‐1β), as well as tumor necrosis factor‐α (TNF‐α), interleukin‐6 (IL‐6), nitric oxide (NO), and reactive oxygen species (ROS) [93]. Moreover, it also induces dysfunction in mitochondrial membrane potential [94]. As a result, antihistamines impact the survival of dopaminergic neurons and mitigate PD symptoms by controlling oxidative stress and suppressing microglia‐induced inflammation. Furthermore, it is noteworthy that SN dopaminergic neurons exhibit a heightened sensitivity to neurotoxicity induced by histamine in comparison to other regions within the basal ganglia [16]. This observation suggests that histamine possesses a specific propensity for inducing neurotoxic effects specifically on dopaminergic neurons. It is plausible to consider that inflammatory mechanisms, which are modulated by histamine, may significantly contribute to the pathological alterations responsible for the damage inflicted upon dopaminergic neurons subsequent to histamine administration.

Regarding the role of histamine receptors in PD pathology, numerous expression studies indicate that H1R affects motor behavior in animals by contributing to the inflammatory response of PD [60, 81, 95]. At the early stage of PD development, the increase in brain histamine levels can exacerbate the loss of dopaminergic neurons in the SN of PD rats induced by 6‐hydroxydopamine (6‐OHDA), which is mediated by the H1R. The administration of the histamine synthetase inhibitor known as alpha‐fluoromethylhistidine (α‐FMH) resulted in a notable delay in the degeneration of dopaminergic neurons caused by 6‐OHDA. Additionally, it effectively mitigated the aberrant turning behavior observed in rats with PD [15]. Moreover, the introduction of histamine into the SN led to the stimulation of microglia, a process that is also mediated by the activation of H1R. This activation process results in dopamine neurotoxicity in adult mice in vivo. This neurotoxicity is dependent on the signaling pathways involving NADPH oxidase 1 (NOX1) and is facilitated by microglial phagocytosis mediated by phosphatidylserine (PS) [81]. In addition, the activation of microglia in animal models of PD leads to phagocytosis, ultimately causing neuronal death. The use of interventions at different phases of microglial phagocytosis has the potential to mitigate neuronal death [96]. Blocking the phagocytosis process by binding PS to annexin V is effective in recovering from the histamine‐induced dopamine toxicity because the “eat me” signal initiated by PS exposure is reversible [81]. Therefore, the inhibition of endogenous histamine production can prevent dopaminergic neuronal degeneration [15]. Meanwhile, ebastine, an H1R antagonist, and levocetirizine, a non‐sedating antihistamine, improved haloperidol‐induced motor impairment in PD mice, reversing their cataleptic state through improved muscle strength, walking balance, and locomotor activity [82]. The action is supported by the observation that the administration of histamine directly to the N27 rat dopamine cell line led to a modest decrease in the viability of the induced cells [81]. This implies that histamine may bypass the “primary phagocytosis” of N9 microglia and trigger the loss of dopaminergic cells by inducing soluble and contact factors, but it is important to do additional research to explore the potential involvement of histamine in causing dopamine‐induced cell loss.

Numerous studies have investigated the affinity of H3R for various GPCRs, ion channels, transporters, and enzymes in the SN [70, 97, 98]. The activation of H3R at the terminal of GABAergic input neurons located in the SNr results in membrane potential hyperpolarization through the downstream coupled GPCR and ion channels, which ultimately decreases the release of GABA [56, 70, 84]. This leads to enhanced excitability of dopaminergic neurons present in SNpc, thereby alleviating the pathological progression of PD. However, there is a consistent reduction in the mRNA expression of H3R in PD, which is specifically localized and detected using immunocytochemistry in the large pigmented neurons of the SNpc [83]. This decrease could potentially enhance the excitability of dopaminergic neurons in the SNpc. Therefore, the heterogeneity of H3R distribution determines the opposite effect in SN. Nonetheless, these studies concur that targeting H3R is beneficial for mitigating the pathological advancement of PD.

H4R activation in the basal ganglia impacts the survival of dopaminergic neurons and symptoms of PD by regulating oxidative stress and inflammation mediated by microglia [31, 99]. H4R mRNA is expressed in rat brain endothelial cells, where it regulates the permeability of the blood–brain barrier (BBB) [100]. Therefore, BBB integrity dysfunction mediated by H4R is considered one of the pathological processes in PD. In addition, in an ischemic model of severe BBB dysfunction, long‐term use of the H4R antagonist JNJ 7777120 was found to reduce ischemic neuronal damage and improve sensorimotor function [101]. Moreover, the H4R mRNA levels in the SN showed a significant increase both in PD patients and rotenone‐induced PD models, and the intracerebellar administration of JNJ 7777120 decreased the pro‐inflammatory phenotype of microglia, reduced α‐synuclein accumulation in the SN and striatum, and attenuated the degeneration of SN dopaminergic neurons in PD model rats [31]. According to an in vivo study, the administration of the H4R antagonist JNJ7777120 has been found to mitigate decreases in dopamine concentrations and restore the equilibrium of 5‐hydroxytryptamine (5‐HT) levels together with its primary metabolite, 5‐hydroxyindoleacetic acid [30]. In addition, the same H4R antagonist preserved levels of glutamine and acetylcholine in the PD mice model [30]. These findings suggest that targeting H4R could be a promising therapeutic target for the movement disorders of PD.

### 4.2 Histamine in the Striatum

The striatum, the largest sub‐nucleus within the basal ganglia, assumes a crucial function in regulating both motor behavior and cognitive function [51, 68]. The striatum receives considerable histaminergic input. Histaminergic neurons project to various parts of the brain, including the striatum. Histamine is released in the striatum by a varicose structure formed by neuronal endings. H1R, H2R, and H3R are co‐expressed with D1R and D2R in the SPNs of the striatum [11, 69, 76]. 95% of striatal neurons are GABAergic SPNs. The SPNs expressing D1R and D2R represent the beginning of the direct and indirect pathways, respectively. The remaining 5% of neurons are cholinergic and GABAergic interneurons that regulate local striatal activity [68]. In the striatum, histaminergic neurons regulate their input by acting on H1R and H2R located in indirect pathway‐striatal projection neurons (d‐SPNs) and i‐SPNs, respectively [11, 76]. H3R can form a heterodimer with both D1R and D2R. Nevertheless, dimerization with D2R leads to a decrease in dopamine's affinity for the receptor, whereas the formation of a heterodimer with D1R prevents cyclic adenosine monophosphate (cAMP) production when D1R is activated. Consequently, stimulation of H3R decreases the overactivity of the indirect pathway similar to the stimulation of D2R, which potentially has positive consequences [69]. Consistently, clinical studies have demonstrated the potential of combining dopamine agonists with the H3R antagonist thiopropionamide to enhance motor function in patients with PD [102]. Therefore, histamine serves as a significant extra‐striatal regulator that actively modulates the functioning of the basal ganglia and plays a role in the development and manifestation of basal ganglia disorders.

On the other hand, histamine can regulate the physiological activity of striatal neural circuits and improve LID. The expression of H2R is prominent in both the striatum, which serves as the input area, and the GP and SN, which function as the output regions inside the basal ganglia. This expression is particularly notable in the striatal pathway, which consists of GABAergic projection neurons [103]. By reducing striatal acetylcholine levels, it can modulate the activation of the D1R‐mediated direct pathway [104]. Long‐term use of the H2R antagonist ranitidine can block the activation of the direct pathway mediated by the D1R, which can lead to the alleviation of the symptoms of LID [85]. Consistently, research has demonstrated that the use of H2R antagonists effectively reduces overall motor deficits by decreasing the excitability of cholinergic interneurons in the dorsolateral striatum, which becomes heightened during the pathology of LID [86]. Moreover, previous research has indicated that H2R antagonists may ameliorate motor impairments through the normalization of G‐protein‐coupled receptor kinases 3 (GRK3), extracellular signal‐regulated kinase activation, and FosB levels in the striatum [87]. Furthermore, the interaction of H3R and D1R has shown that prolonged co‐administration of an H3R agonist and levodopa can significantly reduce involuntary limb, mouth, and tongue movements compared with levodopa alone. For instance, extended usage of immepip (a type of H3R agonist) can reverse and normalize the rise in GABA and glutamate levels found in striatal dialysate caused by prolonged levodopa administration [88]. In PD model rats, the H3R agonist impipride has the potential to enhance the expression of dopamine D1R mRNA in the striatum [89].

### 4.3 Histamine in the Subthalamic Nucleus

As the sole nucleus of excitatory glutamatergic neurons in the basal ganglia circuitry, the STN plays an essential role in the indirect pathway. It forms a hyper‐direct pathway with the cortex, which is considered the pacemaker for the entire basal ganglia [4, 105]. In the pathogenesis of PD, it is commonly accepted that motor impairments arise from an asymmetry in the functioning of the direct and indirect pathways within the basal ganglia circuit, with a particular emphasis on the STN [106]. Consequently, targeting key nodes of the STN has become a favored approach for deep brain stimulation (DBS) in treating PD [107]. The STN exhibits three firing patterns, with the majority of STN neurons demonstrating tonic firing and only a small proportion exhibiting rhythmic and irregular firing [108]. One crucial electrophysiological marker of PD is the notable elevation found in burst and irregular firing patterns of STN neurons. This phenomenon has been directly associated with locomotor impairments in individuals with PD [109].

The STN receives histamine afferents from the TMN. In the clinical progression of PD, there is a significant occurrence of apoptosis in dopaminergic neurons located in the SNpc. This event leads to a substantial rise in the concentration of histamine and the density of histaminergic fibers inside the STN. The administration of a histamine medication injection into the STN of rats with an apomorphine‐induced model of PD resulted in a substantial enhancement in their rotational behavior. This unforeseen improvement caused by histamine is mediated through the activation of H2R rather than H1R, and involves their downstream hyperpolarization‐activated cyclic nucleotide‐gated (HCN) channels to regulate the firing pattern of STN neurons. Furthermore, the overexpression of hyperpolarization‐activated cyclic nucleotide‐gated channel 2 (HCN2) channels in the STN neurons of the PD rat model can regulate the firing patterns of neurons and improve motor symptoms [66]. This suggests that the expression of HCN channels in STN neurons is closely associated with the regulation of neuronal firing patterns and may even be linked to motor symptoms related to PD. Moreover, the consistent modulation of STN burst firing, achieved by the utilization of diverse polarity of DBS, will result in a corresponding reduction or rise in movement abnormalities [109]. On the other hand, DBS normalizes STN neuronal firing patterns by increasing endogenous histamine release, activating H2R‐coupled HCN2 channels, and improving PD motor deficits by inhibiting excessive beta‐network oscillations [66]. The augmentation of brain histamine levels may serve as a crucial mechanism to alleviate motor symptoms, providing a novel PD treatment approach. Therefore, regulating the firing pattern of STN neurons could be an effective strategy for managing the symptoms of movement in PD [105, 107]. In addition, the HCN channel has a distinct expression profile and significantly affects neuronal excitability, rhythmic activity, and resting membrane potential [110]. All four HCN channels, including those in the STN, are widely expressed in the basal ganglia nuclei of mammalian brains [111]. Thus, drugs that target individual HCN isoforms and/or their auxiliary subunit, TRIP8b, in the STN may be valuable targets for the treatment of PD [80].

### 4.4 Histamine in the Lateral Globus Pallidus

The GPe, or LGP, situated centrally within the basal ganglia pathway, serves as a crucial intermediary hub for the indirect pathways in the basal ganglia system [112]. It receives input from various sources, such as the cortical, striatal, and STN, and subsequently transmits its neural fibers to other interconnected nuclei within the basal ganglia circuit, including the STN and SNr [4]. Functionally, the LGP and its downstream STN form the “pacemaker” of the basal ganglia circuit, and this functional association significantly affects the dysfunction of the basal ganglia [12, 113]. In the context of PD pathology, including the loss of dopaminergic neurons, β‐oscillations that arise from cortical microcircuits within the basal ganglia are enhanced and regulated by LGP self‐inhibition. This self‐inhibition plays a crucial role in controlling the balance between upper and lower β‐oscillations. Increased self‐inhibition enhances upper β‐oscillations resulting from cortical rhythms. In contrast, decreased self‐inhibition enhances lower β‐oscillations caused by increased basal ganglia excitability [114, 115, 116]. In addition, neuronal heterogeneity leads to differences in the role of the LGP in PD symptoms. LGP contains neurons that express the biomarkers PV and Lim‐homeobox 6 (Lhx6), respectively [12, 117]. Optogenetic activation of PV neurons in LGP and inhibition of Lhx6‐expressing neurons in LGP using DBS effectively alleviated resting and motor delays in dopamine‐depleted mice within hours of stimulation. This confirms the potential for specific targeted intervention in PD [118]. Therefore, the neuronal diversity in LGP presents numerous avenues for addressing neurological disorders.

The LGP receives histaminergic projections from the TMN, and histamine plays a role in both motor regulation and PD pathology through actions on LGP neurons via pre‐ and post‐synaptic receptors [12, 92]. Intraventricular injection of histamine in rats produces transient locomotor enhancement followed by locomotor inhibition similar to walking. Post‐synaptic H1R antagonists effectively inhibit the locomotor augmentation caused by histamine, whereas pre‐synaptic H3R antagonists diminish the following locomotor inhibition. This suggests that H1R mediates histamine‐induced motor enhancement while H3R mediates subsequent motor deficiency [90]. Similarly, mice lacking H3R showed a decrease in ambulatory movements [119], and H1R agonists strengthen the induction and expression of caffeine‐induced exercise sensitization, whereas their antagonists diminish this effect [91]. In addition, intraventricular administration of α‐FMH, an irreversible inhibitor of histidine decarboxylase, along with H1R and H2R antagonists and H3R agonists, has been shown to enhance motor behavior in rats with PD [92]. Moreover, endogenous histaminergic innervation in the LGP facilitates bidirectional motor regulation via both its pre‐synaptic and post‐synaptic receptors. Histamine regulates the inherent excitability of PV neurons in LGP via post‐synaptic H1R and H2R, and pre‐synaptic H3R negatively governs its impact. The convergence of pre‐synaptic and post‐synaptic impacts occurs at PV neurons, which subsequently affects the operation of indirect pathways involved in movement and plays a role in regulating motor behavior [12]. In addition, the regulatory effects of histamine on LGP PV neurons are mediated by the TWIK‐related potassium channel‐1 (TREK‐1) and the small‐conductance calcium‐activated potassium channel 3 (SK3), which are downstream of the H1R receptor. Furthermore, the HCN2, downstream of the H2R receptor, also plays a role in mediating these effects. This implies that the strategic focus on these pathways may be essential in the management of symptoms associated with PD [12, 120, 121].

### 4.5 Histamine in the Entopeduncular Nucleus

The EPN in rodents or the GPi in primates is located caudomedial to the striatum, which, together with the adjacent SNr, serves as the main output nucleus mass of the basal ganglia. As one of the two main output structures of the basal ganglia, the EPN plays a crucial role in the direct and indirect pathways of the basal ganglia thalamocortical circuit [122]. In the pathological process of PD, significant dopamine depletion in the basal ganglia affects EPN neuronal firing activity, resulting in increased firing irregularity and several bursting neurons [123]. In patients with PD and experimental models, the STN exhibits hyperactivity and amplifies the excitatory glutamatergic input to the GPi/EPN, which also displays hyperactivity [106, 123]. In addition, decreased dopamine levels promote heightened oscillatory activity and synchronization among and between the basal ganglia nuclei, such as the subthalamic nucleus, GPi/EPN, and the cerebral cortex, specifically in beta frequency bands [124]. The aforementioned alterations pertain to the motor symptoms observed in individuals diagnosed with PD, indicating that manipulating the electrical properties of the GPi/EPN might potentially be a promising alternative approach for mitigating symptoms associated with PD.

Immunohistochemical and receptor autoradiographic studies have revealed moderately dense histaminergic fibers on the EPN in rats and guinea pigs. Additionally, the distribution of histamine receptors on the GPi has been identified in postmortem human and rhesus monkey specimens [125, 126, 127]. In the EPN, PV‐positive neurons are mainly concentrated in the posterior two‐thirds of the EPN, while somatostatin (Sst) neurons are found in the rostral/anterior half and the shell region of the EPN [128, 129, 130]. In mice that were subjected to 6‐OHDA‐induced damage, the EPN exhibited elevated histamine levels. This compensatory response to the injury subsequently triggered the activation of PV neurons inside the EPN. These PV neurons are transmitted to the thalamus; therefore, modulating the firing rate of neurons in the EPN. This modulation was mediated by the H2R and its associated HCN2 channel. Meanwhile, the pre‐synaptic H3R of STN neurons feedback regulates this loop, and histamine activates H3R in the axon terminals of STN neurons that project to the EPN, thereby inhibiting PV neurons. Interestingly, activation of both receptors has been shown to improve parkinsonism‐related motor dysfunction in behavioral studies. Among them, the activation or upregulation of H3R on STN neurons that project to the EPN reduced the firing rate but not the firing pattern of PV neurons and improved motor dysfunction. On the other hand, activation of the H2R on PV neurons in the EPN reduces the burst firing pattern. Although both the firing rate and firing pattern of PV neurons are associated with motor deficits in PD, the firing pattern of neurons appears to be more critical [20]. Therefore, targeting H2R and its downstream coupled HCN2 channels in EPN PV neurons and H3R in STN neurons projecting to the EPN may represent potential therapeutic strategies for the clinical treatment of parkinsonism‐related motor dysfunction.

## 5 Conclusion

The central histamine system in the brain is derived from the TMN of the hypothalamus, and its fibers project to almost all the regions of the brain, including the basal ganglia. Under normal physiological conditions, afferent input from histaminergic fibers in the basal ganglia promotes normal motor behavior in animals. In the pathological progression of PD, there is an increase in the concentration of histamine within the basal ganglia. Histamine, via postsynaptic H1R or H4R, plays a significant role in the inflammatory response associated with PD and contributes to the degeneration of dopaminergic neurons by influencing microglial activity in the SNpc. Simultaneously, histamine plays a crucial role in modulating neuronal excitability and firing activity—encompassing both firing rate and pattern—of neurons within the basal ganglia nuclei. This modulation occurs through postsynaptic H1R or H2R receptors, as well as presynaptic H3R receptors and their associated downstream ion channels. Consequently, the influence of histamine results in alterations to motor symptoms and LID observed in animal models of PD. Exploration of the regulatory mechanism of histamine in PD, a basal ganglia‐related disorder, potentially offers hope in alleviating movement disorders associated with PD. The role and regulation of histamine in PD can not only help us understand the occurrence and development of the pathological process of the disease but also has significant implications for drug design and clinical guidance, as well as offering a novel strategy for the clinical relief and treatment of Parkinsonian motor symptoms. The exact mechanisms by which histamine affects PD symptoms are still not fully understood; therefore, further research is required to fully understand the contribution of histamine to the pathogenesis of PD in order to determine its potential as a therapeutic target.

## Author Contributions

Qian‐Xing Zhuang and Ling Shan designed the paper; Hui‐Xian Zhu, Wei‐Wei Lou, Yi‐Miao Jiang, Alina Ciobanu, Chen‐Xin Fang, Cheng‐Ye Liu, Yan‐Li Yang, and Jing‐Yang Cao wrote the paper and designed the figures; and Qian‐Xing Zhuang and Ling Shan reviewed the manuscript. All the authors reviewed and contributed to the final version of the manuscript.

## Conflicts of Interest

The authors declare no conflicts of interest.

## Data Availability

Data sharing does not apply to this article, and this research does not involve the analysis and innovation of new data.
